# Activity and cryo-EM structure of the polymerase domain of the human norovirus ProPol precursor

**DOI:** 10.1128/jvi.01193-24

**Published:** 2024-10-30

**Authors:** Alice M. McSweeney, Alice-Roza Eruera, Geena M. McKenzie-Goldsmith, James C. Bouwer, Simon H. J. Brown, Louise A. Stubbing, Jonathan G. Hubert, Rinu Shrestha, Kevin J. Sparrow, Margaret A. Brimble, Lawrence D. Harris, Gary B. Evans, Mihnea Bostina, Kurt L. Krause, Vernon K. Ward

**Affiliations:** 1 Department of Microbiology and Immunology, School of Biomedical Sciences, University of Otago, Dunedin, New Zealand; 2 Department of Biochemistry, School of Biomedical Sciences, University of Otago, Dunedin, New Zealand; 3 School of Chemistry and Molecular Bioscience, Molecular Horizons, and Australian Research Council Centre for Cryo-electron Microscopy of Membrane Proteins, University of Wollongong, Wollongong, New South Wales, Australia; 4 School of Chemical Sciences, The University of Auckland, Auckland, New Zealand; 5 Ferrier Research Institute, Victoria University of Wellington, Lower Hutt, New Zealand; St. Jude Children's Research Hospital, Memphis, Tennessee, USA

**Keywords:** norovirus, ProPol, polymerase, precursor protein

## Abstract

Human norovirus (HuNV) is a leading cause of acute gastroenteritis worldwide with most infections caused by genogroup I and genogroup II (GII) viruses. Replication of HuNV generates both precursor and mature proteins during processing of the viral polyprotein that are essential to the viral lifecycle. One such precursor is protease-polymerase (ProPol), a multi-functional enzyme comprised of the norovirus protease and polymerase proteins. This work investigated HuNV ProPol by determining the *de novo* polymerase activity, protein structure, and antiviral inhibition profile. The GII ProPol *de novo* enzymatic efficiencies (*k*_cat_/*K*_m_) for RNA templates and ribonucleotides were equal or superior to those of mature GII Pol on all templates measured. Furthermore, GII ProPol was the only enzyme form active on a poly(A) template. The first structure of the polymerase domain of HuNV ProPol in the unliganded state was determined by cryo-electron microscopy at a resolution of 2.6 Å. The active site and overall architecture of ProPol are similar to those of mature Pol. In addition, both galidesivir triphosphate and PPNDS inhibited polymerase activity of GII ProPol, with respective half-maximal inhibitory concentration (IC_50_) values of 247.5 µM and 3.8 µM. In both instances, the IC_50_ obtained with ProPol was greater than that of mature Pol, indicating that ProPol can exhibit different responses to antivirals. This study provides evidence that HuNV ProPol possesses overlapping and unique enzyme properties compared with mature Pol and will aid our understanding of the replication cycle of the virus.

**IMPORTANCE:**

Despite human norovirus (HuNV) being a leading cause of acute gastroenteritis, the molecular mechanisms surrounding replication are not well understood. Reports have shown that HuNV replication generates precursor proteins from the viral polyprotein, one of which is the protease-polymerase (ProPol). This precursor is important for viral replication; however, the polymerase activity and structural differences between the precursor and mature forms of the polymerase remain to be determined. We show that substrate specificity and polymerase activity of ProPol overlap with, but is distinct from, the mature polymerase. We employ cryo-electron microscopy to resolve the first structure of the polymerase domain of ProPol. This shows a polymerase architecture similar to mature Pol, indicating that the interaction of the precursor with substrates likely defines its activity. We also show that ProPol responds differently to antivirals than mature polymerase. Altogether, these findings enhance our understanding of the function of the important norovirus ProPol precursor.

## INTRODUCTION

The norovirus genus in the *Caliciviridae* family is classified into at least 10 genogroups (GI-GX) infecting humans, mice, cattle, pigs, and dogs (1–5). Human norovirus (HuNV) is a leading cause of acute gastroenteritis worldwide, with an estimated 685 million cases each year, the majority of which are caused by genogroup I (GI) and genogroup II (GII) viruses (6, 7). Despite the significant burden imposed by these viruses, there are currently no approved vaccines or antivirals available to treat GI or GII HuNV. Advances in culturing of the virus in stem cell-derived human enteroids and zebrafish larvae have increased our ability to directly study HuNV (8–10). However, the historical lack of culture systems has limited our understanding of the replication mechanisms of noroviruses.

HuNV consists of a positive-sense single-stranded ~7.5 kb RNA genome that is covalently linked to viral protein genome-linked (VPg) at the 5′-end and polyadenylated at the 3′-end. The genome is organized into three open-reading frames (ORF), which give rise to the non-structural (ORF1) and structural (ORF2 and ORF3) proteins. ORF1 is translated as a single polyprotein that is processed into the mature non-structural proteins: NS1-2 (N-term), NS3 (NTPase), NS4, NS5 (VPg), NS6 (protease, Pro), and NS7 (polymerase, Pol). Because the protease cleavage sites between these mature proteins are recognized with varying efficiencies, several precursor proteins are generated during replication, one of which is the protease-polymerase (ProPol) protein (11, 12).

Precursor proteins often perform alternate functions to mature proteins, thereby helping to maximize the limited coding capacity of the viral genome (13–16). Norovirus ProPol possesses protease, polymerase, and nucleotidylylation activities that are all essential for viral replication (11, 17–19). Enzymatically active ProPol proteins have been characterized in other caliciviruses including rabbit hemorrhagic disease virus (RHDV) (lagovirus) and feline calicivirus (vesivirus), indicating that the presence of the precursor protein is conserved in the *Caliciviridae* family (20, 21). An analogous but well-characterized protein, 3CD, is produced by viruses in the *Picornaviridae* family, although, 3CD is only active as a protease and does not possess polymerase activity (16).

As a protease, norovirus ProPol displays alternate substrate preferences to mature Pro, likely contributing to early and late polyprotein processing (22, 23). HuNV Pro has been shown to bind RNA sequences representing the 5′ or 3′ genome ends (24). This interaction inhibits protease activity of both Pro and ProPol, with the most potent inhibition occurring with longer lengths of RNA (24).

Norovirus ProPol and Pol are both active polymerase enzymes and can perform RNA synthesis in either a primed or *de novo* manner (17, 25, 26). Mature HuNV Pol has been shown to possess *de novo* polymerase activity on a poly(C) RNA template but was inactive with other homopolymeric templates (26). Both HuNV ProPol and Pol can perform nucleotidylylation of VPg *in vitro*; however, ProPol is 100-fold more active than mature Pol (18). Similarly, RHDV ProPol is up to 13-fold more active than Pol in *in vitro* nucleotidylylation assays (20), further illustrating functional differences between mature and precursor polymerase enzymes within the *Caliciviridae* family.

The closest relevant atomic structure to ProPol is a 3.4 Å resolution crystal structure of a mutant poliovirus 3CD (PDB code: 2IJD) (27). However, the proteins are functionally distinct and there is limited amino acid sequence similarity between the two enzymes (~30%); therefore the poliovirus 3CD structure is of limited utility for exploring norovirus ProPol function and activity. Structural studies on norovirus enzymes have focused on mature Pro and Pol (28–32). Indeed, several crystal structures of HuNV mature Pol have been solved, including ligand-free and drug-bound polymerases (PDB: 4LQ3, 4NRT, and 5TSN) (33–35). These structures reveal that HuNV Pol is highly structurally conserved with other Group 1 RNA polymerases, possessing a semi-closed right-hand architecture with thumb, palm, and fingers domains (31, 36). The fingers and thumb domains are connected by a series of loop and turn structures, which form a bridge, and the palm domain is lined with well-established motifs designated A-G, which are important for polymerase activity (37). Interestingly, the currently resolved structures of mature Pol and VPg are incompatible for nucleotidylylation without a major conformational change in one or both proteins, although ProPol is primarily responsible for nucleotidylylation (18, 38). In the absence of a ProPol structure, inhibitor design and screening studies necessarily resort to using mature Pol structures as a model (31, 36, 39, 40).

This work aimed to expand our understanding of the polymerase activity of HuNV ProPol, by investigating the ability of different RNA substrates to act as polymerase templates, determine a 3D structure of the polymerase precursor, and measure inhibition by select antivirals. Our results demonstrate that GII ProPol displays polymerase activity with a broader range of RNA templates and has kinetic parameters that are equal or superior to GII Pol, except for the *k*_cat_ for GTP. We present the first structure of the polymerase domain of GII.4 Sydney 2012 HuNV ProPol without substrate, solved by cryo-electron microscopy (cryo-EM). Finally, we show that antiviral compounds targeting mature Pol can also inhibit ProPol, albeit with different efficacies. Overall, while there are similarities between ProPol and Pol, the enzymes have distinct polymerase activities that are essential to the replication of the virus.

## RESULTS

### Norovirus ProPol initiates *de novo* RNA synthesis on homopolymeric RNA templates

**Fig 1:**
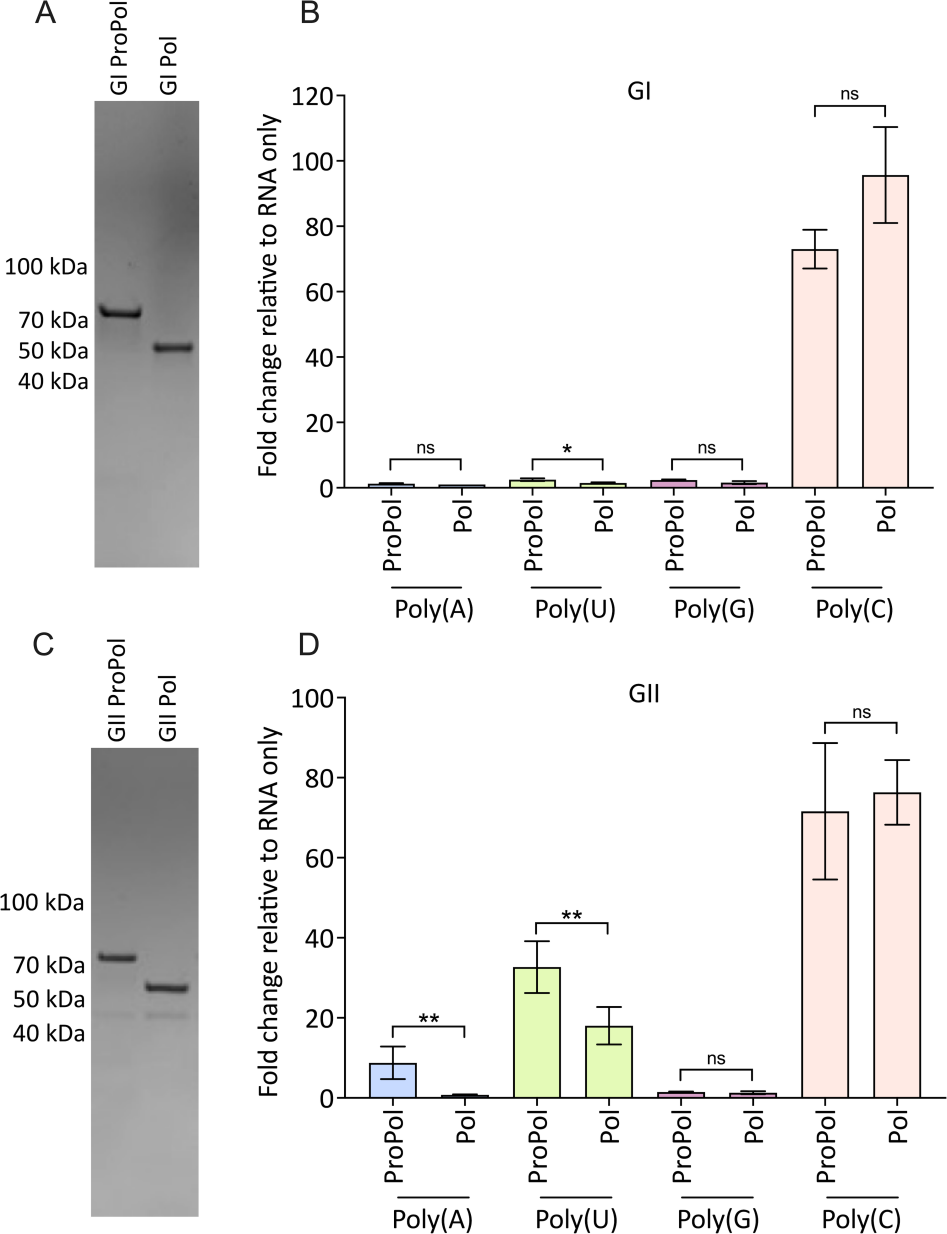
*De novo* polymerase activity on homopolymeric RNA templates. (**A and C**) SDS-PAGE analysis of purified GI and GII ProPol and Pol proteins. (**B and D**) Polymerase activity in the presence of 1 µM enzyme, 20 ng/µL template, and 0.5 mM nucleotide triphosphate (NTP) for 60 min. Fold change relative to an RNA-only control was calculated for each template. Results represent the mean and SD of three independent reactions. Data were analyzed by two-tailed unpaired *t*-test, **P < 0.01, *P < 0.05, and not significant (ns) = *P* > 0.05.

ProPol and Pol proteins from GI Southampton and GII.4 Sydney 2012 HuNVs were expressed using the SUMO Cloning and Expression System (Lucigen) to obtain native ProPol and Pol without additional N- or C-terminal amino acids, at ~76 kDa and ~57 kDa, respectively (Fig. 1A and C). To investigate *de novo* polymerase activity on homopolymeric RNA templates, 1 µM of either ProPol or Pol from GI and GII noroviruses was incubated in a standard polymerase reaction for 60 min. The fold change in relative fluorescent units (RFU) relative to an RNA-only control was calculated to measure formation of dsRNA.

Both the GI and GII versions of ProPol and Pol were active on a poly(C) RNA template, with no statistical significance between the two enzymes (Fig. 1B and D). The GI enzymes showed negligible activity on poly(A), poly(U), and poly(G) templates that was insufficient for further kinetic analysis (Fig. 1B). In contrast, GII ProPol and Pol were both active with a poly(U) template, with a 32-fold and 18-fold increase, respectively, over RNA only (Fig. 1D). Only GII ProPol was active with a poly(A) template, GII Pol did not show activity on a poly(A) template. As with the GI proteins, GII ProPol and Pol did not show observable activity with a poly(G) template (Fig. 1D). Overall, this shows that GII ProPol and Pol have different template preferences to GI enzymes. In addition, unlike the mature GII Pol, the GII ProPol has activity on a poly(A) template.

The importance of linkage of Pro to Pol in the form of ProPol upon polymerase activity was examined, with addition of GII Pro to Pol enhancing polymerase activity on a poly(U) template 25-fold over RNA only (Fig. S1). However, this is less than the fold increase observed with GII ProPol indicating that linkage in the form of ProPol is more active than a combination of independent enzymes on this template. Addition of GII Pro to Pol with a poly(A) template did not induce any changes in polymerase activity (Fig. S1).

### Kinetic analysis of norovirus ProPol and Pol

To further characterize the enzymatic activity of GI and GII HuNV ProPol and Pol, the linear phase of each enzyme-substrate pair was determined, and the steady-state kinetics was established.

**Fig 2:**
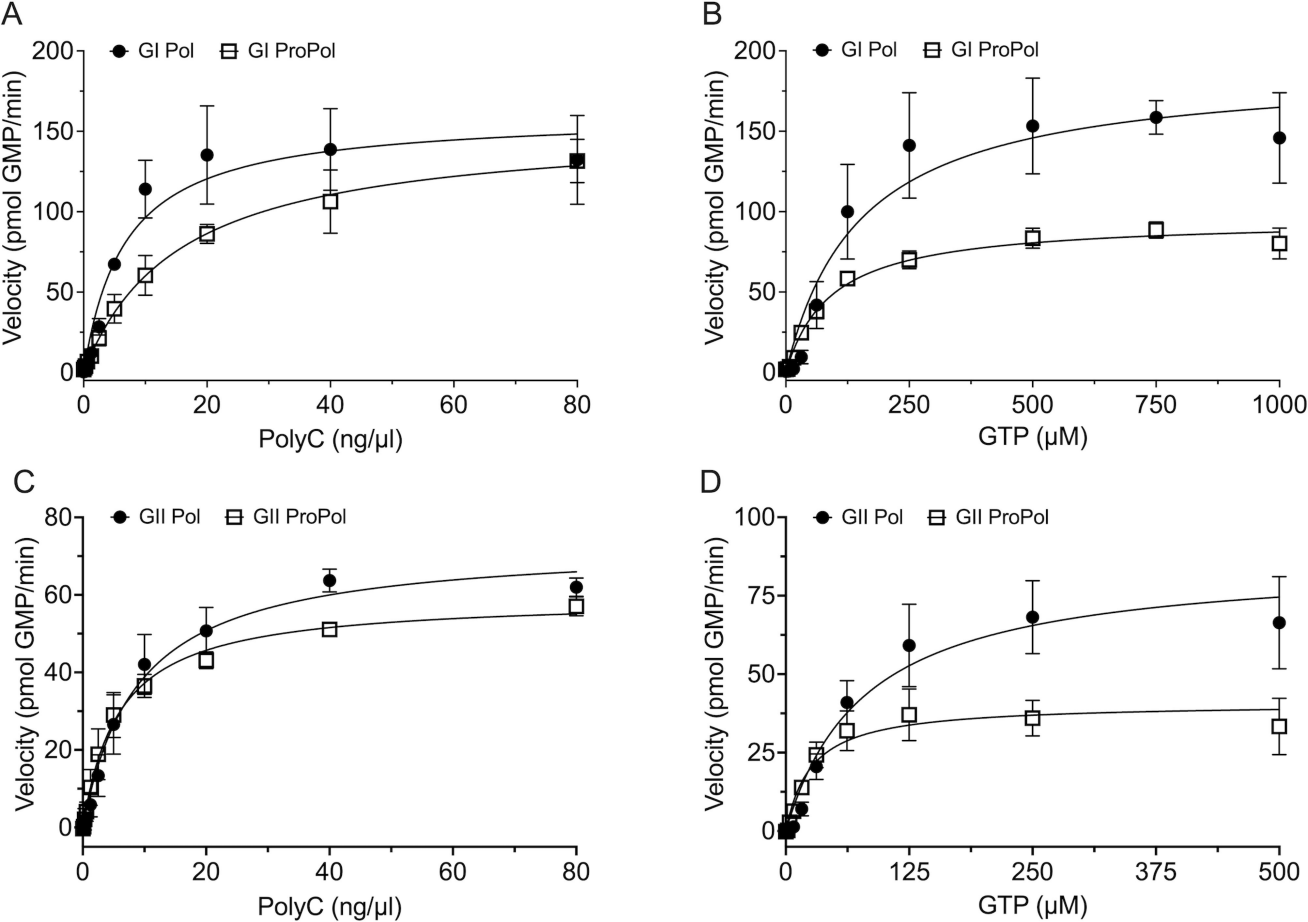
Kinetics of poly(C) and GTP substrate utilization. Purified GI ProPol (250 nM) and Pol (75 nM) or GII ProPol (35 nM) and Pol (35 nM) were used to generate dsRNA in a PicoGreen assay. (**A and C**) Kinetics of poly(C) template utilization was analyzed by titrating template from 0 to 80 ng/µL in the presence of 0.5 mM GTP. (**B and D**) Kinetics of GTP utilization was analyzed by titrating GTP from 0 to 1,000 µM (GI) (**B**) or 500 µM (GII) (**D**) in the presence of 20 ng/µL poly(C). Following a 10-min incubation at 37°C, the reactions were stopped with 10 mM EDTA and dsRNA was quantified using PicoGreen. GI and GII Pol are represented as circles and ProPol as squares. The solid lines show the fit of the data to the Michaelis-Menten equation [*R*^2^ values of GI Pol poly(C) 0.91 and GTP 0.90; GI ProPol poly(C) 0.97 and GTP 0.98; GII Pol poly(C) 0.97 and GTP 0.92; and GII ProPol poly(C) 0.98 and GTP 0.91]. Results represent the mean and SD for three independent experiments with triplicate reactions for each measurement point.

For both poly(C) and GTP, the data could be fit to the Michaelis-Menten model and generated *K*_m_, *k*_cat_, and *k*_cat_/*K*_m_ values (Fig. 2; Table 1). On a poly(C) template, GI Pol, GII ProPol, and GII Pol displayed similar values for *K*_m_, *k*_cat_, and *k*_cat_/*K*_m_ (Table 3). In contrast, there was a trend for higher *K*_m_ at 15.6 ng/µL, lower *k*_cat_ and lower *k*_cat_/*K*_m_ at 0.4 s^−1^ and 0.02 ng/µL^−1^s^−1^, respectively, for GI ProPol with poly(C) compared with the other enzymes (Table 1). With GTP, GII ProPol generated a *K*_m_ of 25.3 µM, approximately threefold lower than the corresponding *K*_m_ for GII Pol of 80.3 µM (Table 1). Although less apparent, the same trend was observed for GI ProPol, with a *K*_m_ of 91.5 μΜ and GI Pol with a *K*_m_ of 150.4 µM. The *k*_cat_ values, 0.24 s^−1^ for GI ProPol and 0.78 s^−1^ for GII ProPol, were lower than those of the Pol enzymes, suggesting Pol turns over molecules of GTP more efficiently (Table 1). The *k*_cat_/*K*_m_ values for GII ProPol and Pol were similar at 0.03 µM^−1^ s^−1^ and 0.02 µM^−1^ s^−1^. However, for GI ProPol, it was 3.8-fold lower than that of GI Pol at 0.01 µM^−1^ s^−1^ (Table 1).

**TABLE 1:** Kinetic parameters with poly(C) template RNA or GTP

	GI ProPol	GI Pol	GII ProPol	GII Pol
*K*_m_ poly(C) (ng/μL)	15.6 (12.0–20.2)^*a*^	6.5 (5.2–8.0)	5.9 (4.7–7.3)	8.8 (6.9–11.3)
*k*_cat_ poly(C) (s^−1^)	0.4 (0.35–0.42)	1.4 (1.3–1.6)	1.1 (1.0–1.2)	1.4 (1.3–1.5)
*k*_cat_/*K*_m_ poly(C) (ng/μL^−1^ s^−1^)	0.02	0.22	0.19	0.16
*K*_m_ GTP (μM)	91.5 (75.1–111.7)	150.4 (97.7–234.9)	25.3 (17.5–38.0)	80.3 (51.6–125.8)
*k*_cat_ GTP (s^−1^)	0.24 (0.22–1.86)	1.7 (1.47–1.96)	0.78 (0.69–0.87)	1.6 (1.40–1.96)
*k*_cat_/*K*_m_ GTP (μM^−1^ s^−1^)	2.6 × 10^−3^	0.01	0.03	0.02

^*a*^ Numbers in parentheses are the 95% confidence interval for the kinetic parameter.

**Fig 3:**
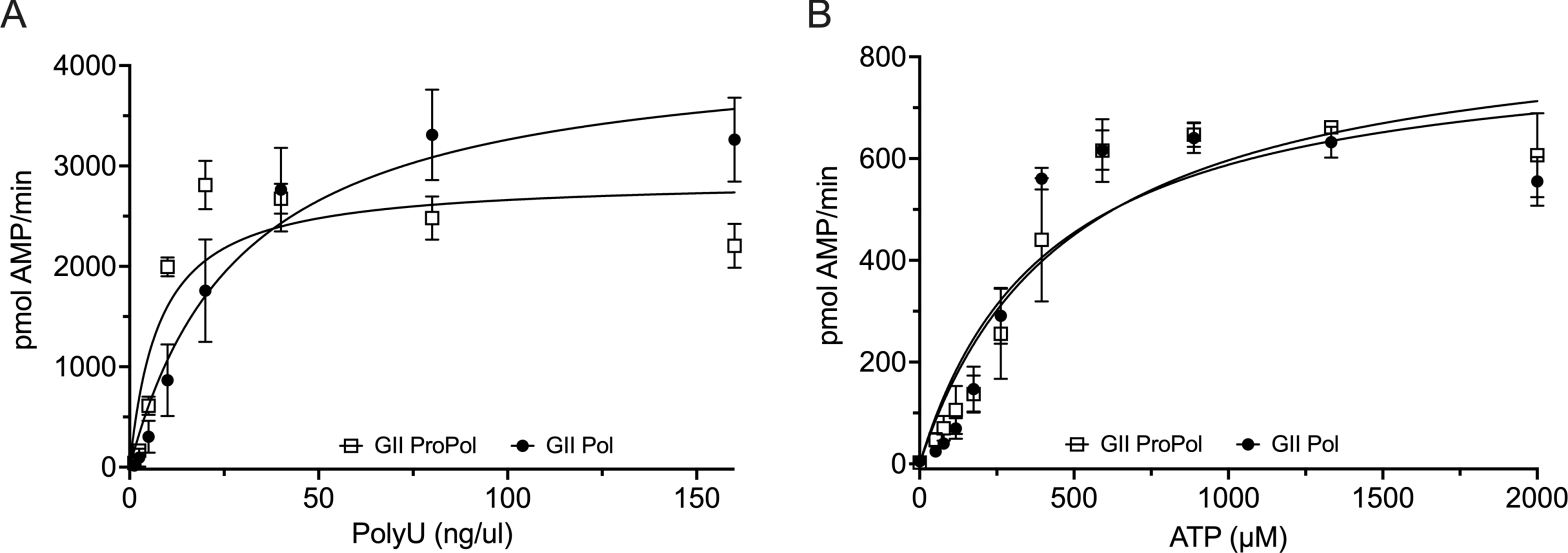
Kinetics of poly(U) and ATP substrate utilization. Purified GII ProPol (1 µM) and Pol (2 µM) were used to generate dsRNA for 20 min in a PicoGreen assay. (**A**) Kinetics of poly(U) template utilization was analyzed by titrating template from 0 to 160 ng/µL in the presence of 0.5 mM ATP. (**B**) Kinetics of ATP utilization was analyzed by titrating ATP from 0 to 2000 µM in the presence of 80 ng/µL poly(U). GII Pol activity is shown as filled circles and ProPol activity as open squares. The solid lines show the fit of the data to the Michaelis-Menten equation [*R*^2^ values of GII Pol poly(U) 0.92 and ATP 0.85; GII ProPol poly(U) 0.80 and ATP 0.90]. Results represent the mean and SD for three independent experiments with triplicate reactions for each measurement point.

Next, the steady-state parameters of GII ProPol and Pol with poly(U) template RNA and ATP were assessed (Fig. 3; Table 2). GII ProPol showed a *K*_m_ for poly(U) of 7.9 ng/µL, 3.7-fold lower than that of GII Pol at 29.6 ng/µL, suggesting an increased affinity for poly(U) by ProPol. At 1.9 s^−1^ and 1.6 s^−1^, the *k*_cat_ values for ProPol and Pol were similar. However, the *k*_cat_/*K*_m_ specificity constant for ProPol, 0.24 ng/µL^−1^ s^−1^, was 5.1-fold higher than that of Pol at 0.047 ng/µL^−1^ s^−1^ for poly(U). There was no difference in the *K*_m_ for ATP between GII ProPol and Pol.

**TABLE 2:** Kinetic parameters with poly(U) template RNA or ATP

	GII ProPol	GII Pol
*K*_m_ poly(U) (ng/μL)	7.9 (4.7–13.2)^*a*^	29.6 (19.7–45.0)
*k*_cat_ poly(U) (s^−1^)	1.9 (1.6–2.2)	1.4 (1.2–1.7)
*k*_cat_/*K*_m_ poly(U) (ng/μL^−1^ s^−1^)	0.24	0.047
*K*_m_ ATP (μM)	484.2 (324.8–734.0)	416.4 (270.0–649.2)
*k*_cat_ ATP (s^−1^)	0.6 (0.5–0.7)	0.3 (0.2–0.33)
*k*_cat_/*K*_m_ ATP (μM^−1^ s^−1^)	1.2 × 10^−3^	7.2 × 10^−4^

^*a*^ Numbers in parentheses are the 95% confidence interval for the kinetic parameter.

**Fig 4:**
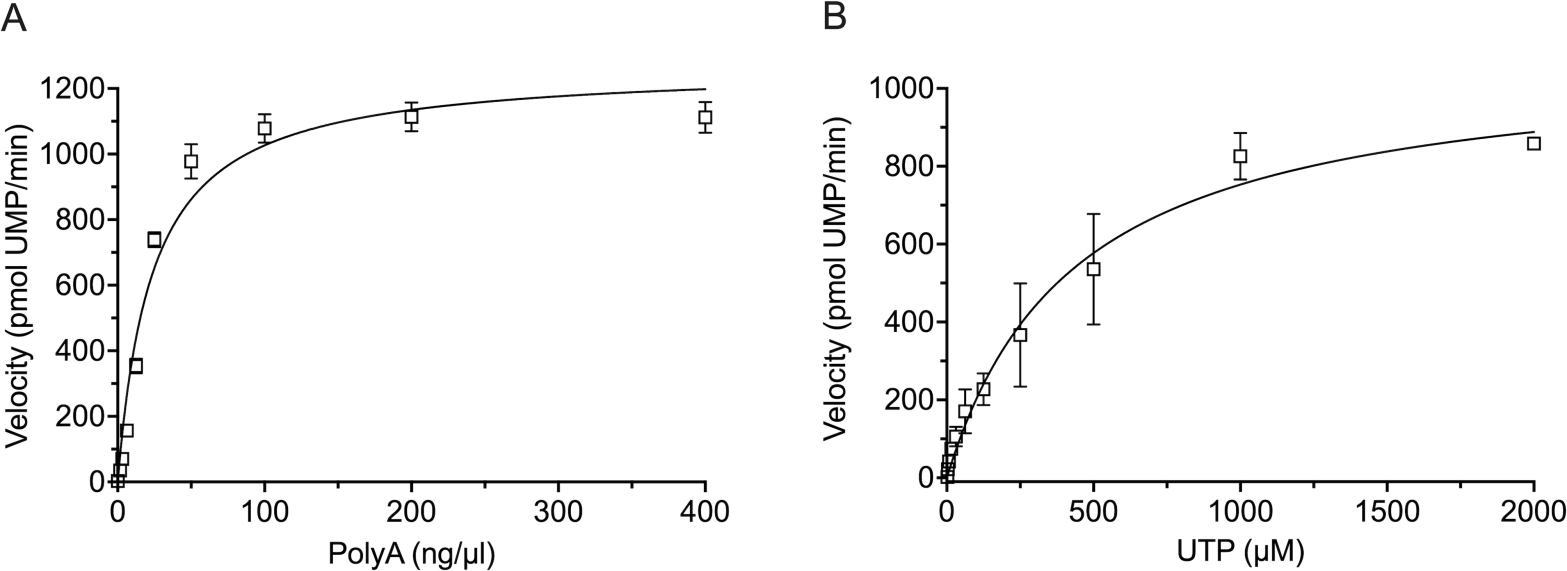
GII ProPol kinetics with poly(A) RNA and UTP. GII ProPol (2 µM) was used to generate dsRNA for 60 min in a PicoGreen assay. (**A**) Kinetics of poly(A) template utilization was analyzed by titrating template from 0 to 400 ng/µL in the presence of 0.5 mM UTP. (**B**) Kinetics of ATP utilization was analyzed by titrating ATP from 0 to 2000 µM in the presence of 20 ng/µL poly(A). Data were fit with the Michaelis-Menten equation [*R*^2^ values of GII ProPol poly(A) 0.97 and UTP 0.96]. Results represent the mean and SD for three independent experiments with triplicate reactions for each measurement point.

Finally, the steady-state kinetics of GII ProPol with a poly(A) template and UTP was tested (Fig. 4); GII Pol was not included as activity with this template could not be detected (Fig. 1). Incubation of GII ProPol with varying concentrations of poly(A) RNA generated a *K*_m_ of 23.6 ng/µL, *k*_cat_ of 0.42 s^−1^, and *k*_cat_/*K*_m_ of 0.017 ng/µL^−1^ s^−1^ (Table 3), while varying UTP concentration generated a *K*_m_ of 437.9 µM, *k*_cat_ of 0.36 s^−1^, and *k*_cat_/*K*_m_ of 8.2 × 10^−4^ µM^−1^ s^−1^ (Table 3). The *k*_cat_/*K*_m_ values show that GII ProPol has the lowest specificity constant for both poly(A) and UTP compared with other substrates tested.

**TABLE 3:** Kinetic parameters with poly(A) template RNA or UTP

	GII ProPol
*K*_m_ poly(A) (ng/μL)	23.6 (19.0–29.3)^*a*^
*k*_cat_ poly(A) (s^−1^)	0.42 (0.39–0.45)
*k*_cat_/*K*_m_ poly(A) (ng/μL^−1^ s^−1^)	0.017
*K*_m_ UTP (μM)	437.9 (317.6–606.8)
*k*_cat_ UTP (s^−1^)	0.36 (0.32–0.41)
*k*_cat_/*K*_m_ UTP (μM^−1^ s^−1^)	8.2 × 10^−4^

^*a*^ Numbers in parentheses are the 95% confidence interval for the kinetic parameter.

### Cryo-EM structure of the polymerase domain of GII.4 ProPol

Kinetic analysis of ProPol and Pol showed that there are differences in substrate specificity and catalytic efficiency, particularly for the GII proteins. This justified an investigation into the structural differences between the two GII enzymes. For high-yield expression, the GII.4 ProPol used in structural experiments was expressed in *T.ni* cells with a N-terminal His_6_ tag. Purified H_6_ProPol retained both protease and polymerase activities indicating that the His_6_ tag does not affect the validity of a resulting structure (Fig. S2).

A cryo-EM map of the polymerase domain of ProPol was solved to a final near-atomic resolution of 2.6 Å. The protease domain (~19 kDa), which is three times smaller than the polymerase domain (~57 kDa) and separated by a flexible region, did not resolve in the map reconstruction, although SDS-PAGE gel analysis confirmed that the ProPol sample was intact at the time of vitrification (Fig. S3). In addition to cryoSPARC, both Relion and manual particle picking were utilized for 2D classification but did not resolve the protease domain. Typical computational techniques used to resolve flexible domains, such as signal subtraction and local resolution masking, were not possible due to the small size of the particles and the lack of specimen contrast in the images. Regardless, a map of the polymerase domain was solved and contains well-resolved, isotropic density for most sidechains and for one of the two metal cations in the active site (Fig. S4). The density quality varies throughout the map with the main chain clear throughout except for occasional breaks within surface loops and near the C-terminus. Regions of high resolution with fully resolved side chains were mixed with regions with less density beyond the beta carbon. The overall density was good and consistent with the reported resolution; however, the “thumb” region from residues 380 to 502 was less well resolved, especially near the C-terminus.

**Fig 5:**
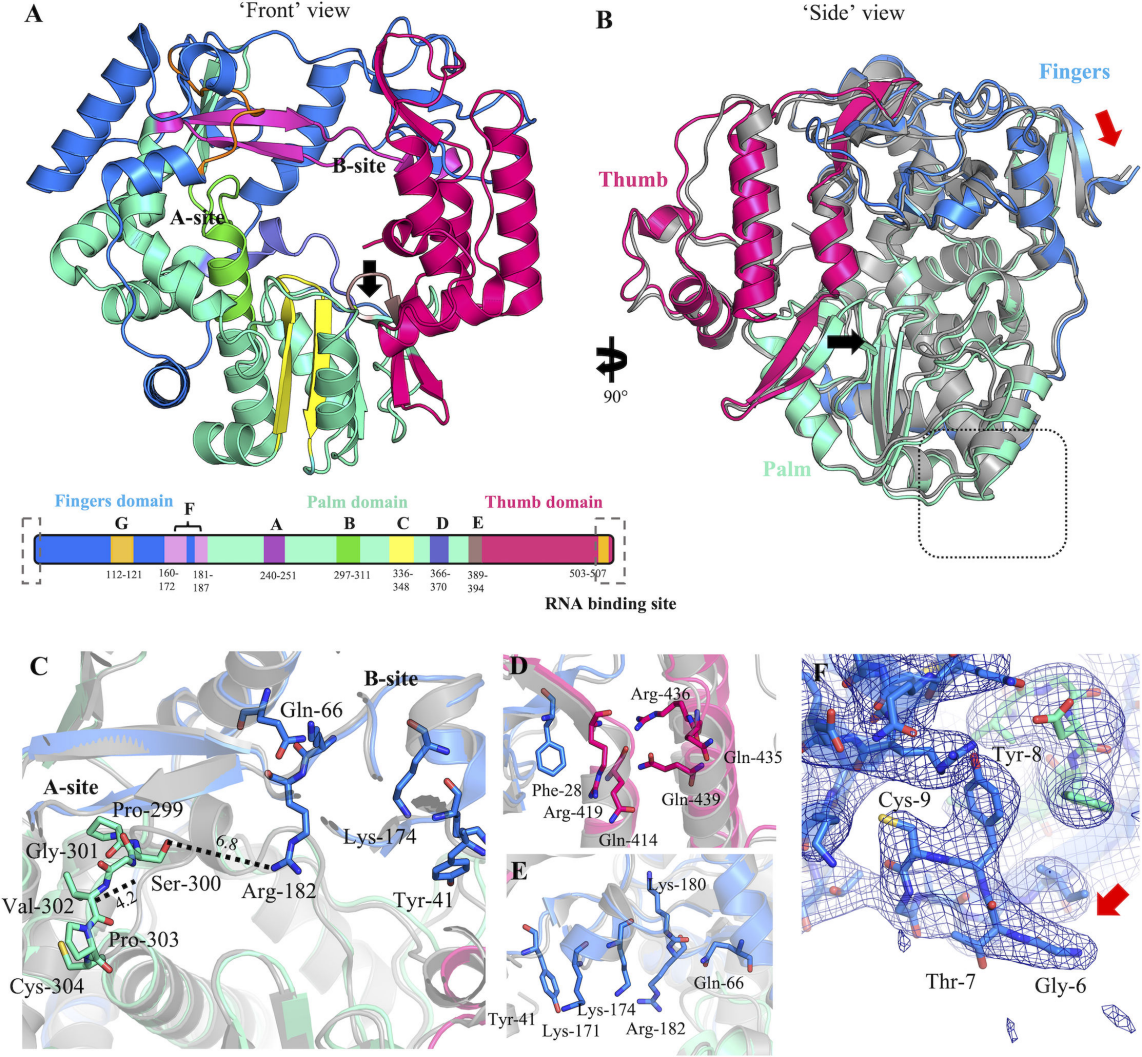
Structural overview of polymerase domain of HuNoV Sydney 2012 ProPol. (**A**) The HuNoV ProPol polymerase domain is represented as a Richardson diagram oriented down the template entry channel and color coded by catalytic motif. Black arrow indicates location of the active site. A domain schematic indicates domain names above and amino acid residue numbers below. Terminal boxes on the schematic indicate regions where density was lacking in the cryo-EM map. Domain regions in schematic are not to scale. (**B**) Side view of Sydney 2012 ProPol (fingers, blue; palm, green; and thumb, pink) overlayed with a ligand-free GII.4 Dresden strain norovirus polymerase crystal structure (gray, PDB: 2B43). Red arrow indicates where the linker between protease and polymerase is located. Black arrow indicates the active site. Dotted box on the palm domain indicates the putative location of the VPg binding site (28). (**C**) A short loop (residues 299–304) within motif B, on the interior of the palm domain, has a shift of ~4.2 Å between ProPol (colored as in A) and the mature polymerase (gray). Distances are indicated by dashed line and measured in angstroms. (**D**) An overlay of the A-site and B-site (**E**) reveals a high degree of structural conservation between mature (Dresden) and precursor (Sydney) Pol. (**F**) Density terminates at Gly-6 of the polymerase domain resulting in a truncated structure composed of a singular polymerase domain.

The precursor possesses the fingers-thumb-palm architecture typical of Group 1 RNA polymerases (Fig. 5A). The fingers domain extends, starting at the N-terminus, from residue 1 to 187, the palm from residues 188 to 394, and the thumb from residue 395 to the C-terminus. When superposed onto the crystal structure of relaxed state unliganded GII.4 Dresden Pol (PDB: 2B43, chain A), there is a root mean square deviation (RMSD) of 1.19 Å across all atom pairs indicating strong structural conservation. In particular, the inner palm domain and lower outer palm domain are highly conserved between precursor and mature polymerases (Fig. 5B), as are the common drug binding A-site and B-site (Fig. 5D and E) (33). However, small shifts in some helices and loops result in modest local structural variations between the mature and precursor polymerases, shifting up to 3.8 Å (Asp-472, fingers), 1.4 Å (Lys-139, thumb), 1.4 Å (Gln-435, fingers), 3.1 Å (Glu-499, fingers), and 2.9 Å (Gly-377, palm) (measured from α-carbon to α-carbon).

Of the conserved catalytic motifs directly involved in polymerization and substrate binding, only a loop within Motif B (Glu-296 to Cys-304) is significantly different between mature and precursor Pol, shifting the main chain by 4.2 Å and bringing Ser-300 to 6.8 Å from Arg-182, the nearest residue of the B-site of the thumb domain, relative to the 6.1 Å distance between the analogous residues reported in the mature Dresden Pol structure (Fig. 5, panel C). This shift results in the loss of a hydrogen bond between Ser-300 and both Asp-247 and Trp-185 as seen in mature Pol. In ProPol instead, we find an interaction, which is most clear in the sharpened map, between the main chain oxygen of Gly-301 and the side chain oxygen of Ser-187 and an interaction between the main chain oxygen of Asp-247 with the main chain nitrogen of Leu-298. The amino acid sequence of this loop is conserved in both Dresden and Sydney 2012 norovirus, and regions of these two structures close to Motif B are structurally conserved. Interestingly, ProPol has a similar Motif B loop arrangement as inhibitor-bound mature polymerases. This loop in mature Dresden Pol adopts the same conformation seen here in unliganded ProPol when the B-site is occupied by a PPNDS ligand, despite the B-site and the ligand being located a substantial distance away from Motif B (Fig. S5).

### Comparison of antivirals targeting HuNV ProPol and Pol

**Fig 6:**
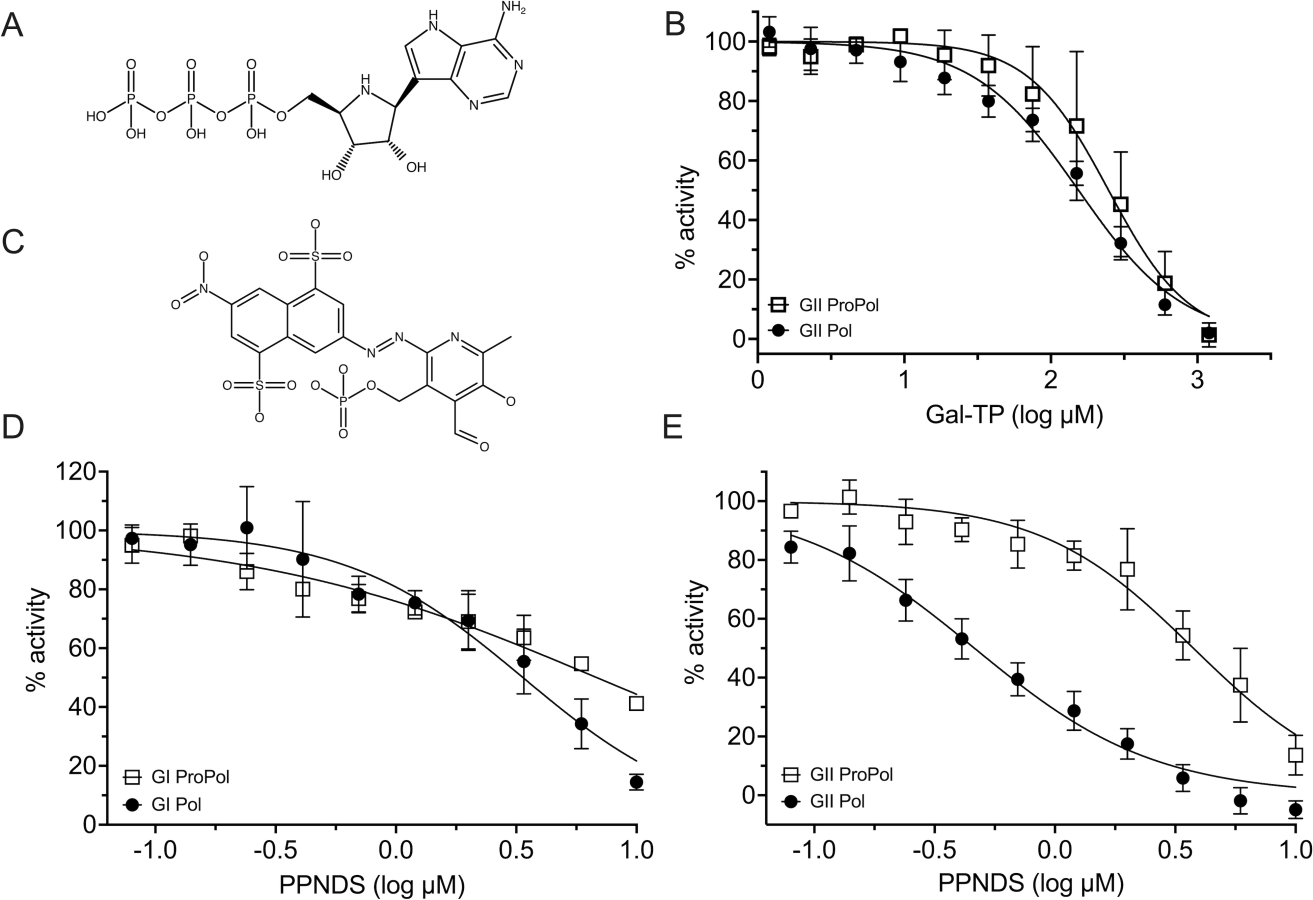
Inhibition by Gal-TP and PPNDS on polymerase activity of GI and GII ProPol and Pol proteins purified from *Escherichia coli*. Chemical structures of (**A**) Gal-TP and (**C**) PPNDS. (**B**) Dose response curve of GII ProPol (1 µM) and GII Pol (2 µM) with Gal-TP and substrate at 8 ng/µL poly(U) template and 400 µM ATP. (**D**) Dose response curves of GI ProPol (250 nM) and GI Pol (75 nM) with PPNDS and substrate at 15 ng/µL poly(C) template and 90 µM GTP or 6 ng/µL poly(C) template and 150 µM for ProPol and Pol, respectively. (**E**) Dose-response curves of GII ProPol (35 nM) and GII Pol (35 nM) with PPNDS and substrate at 6 ng/µL poly(C) template and 25 µM GTP. Data were normalized relative to an enzyme-dimethyl sulfoxide (DMSO) vehicle control. Data represent the mean and SD of three independent experiments with triplicate reactions for each measurement point.

Although there are several norovirus antivirals directed against polymerase activity, it is not known if these compounds are active against precursor proteins, including ProPol (8, 31, 36, 39–44). The effect of galidesivir triphosphate (Gal-TP), a nucleotide analog, and PPNDS, a non-nucleoside inhibitor, on the *de novo* polymerase activity of ProPol and Pol was tested (Fig. 6). Proteins were incubated with an inhibitor before the addition of substrate, and activity was normalized with enzyme only, representing 100% activity and enzyme treated with EDTA, representing 0% activity.

Gal-TP is an ATP analog; therefore, *de novo* polymerase inhibition could only be measured with GII and not GI enzymes, as these did not show activity with a poly(U) template (Fig. 1). In the presence of Gal-TP, polymerase activity of GII ProPol and Pol was inhibited and generated respective half-maximal inhibitory concentration (IC_50_) values of 247.5 µM (95% CI: 201.2–302.9 μM) and 154.3 µM (95% CI: 135.8–175.1 μM) (Fig. 6B). This shows weak inhibition of polymerase activity by Gal-TP, with a subtle but significant 1.6-fold difference between the IC_50_ values.

PPNDS generated a dose-dependent inhibition of GI and GII norovirus ProPol and Pol polymerase activities with a poly(C) RNA template. The IC_50_ values were calculated to be 6.8 µM (95% CI: 5.5–8.9 μM) for GI ProPol and 3.4 µM (95% CI: 2.8–4.1 μM) for GI Pol (Fig. 6D). In comparison, PPNDS was more potent at inhibiting polymerase activity of GII norovirus proteins, with IC_50_ values of 3.8 µM (95% CI: 3.2–4.4 μM) and 0.5 µM (95% CI: 0.4–0.5 μM) with ProPol and Pol, respectively (Fig. 6E).

**Fig 7:**
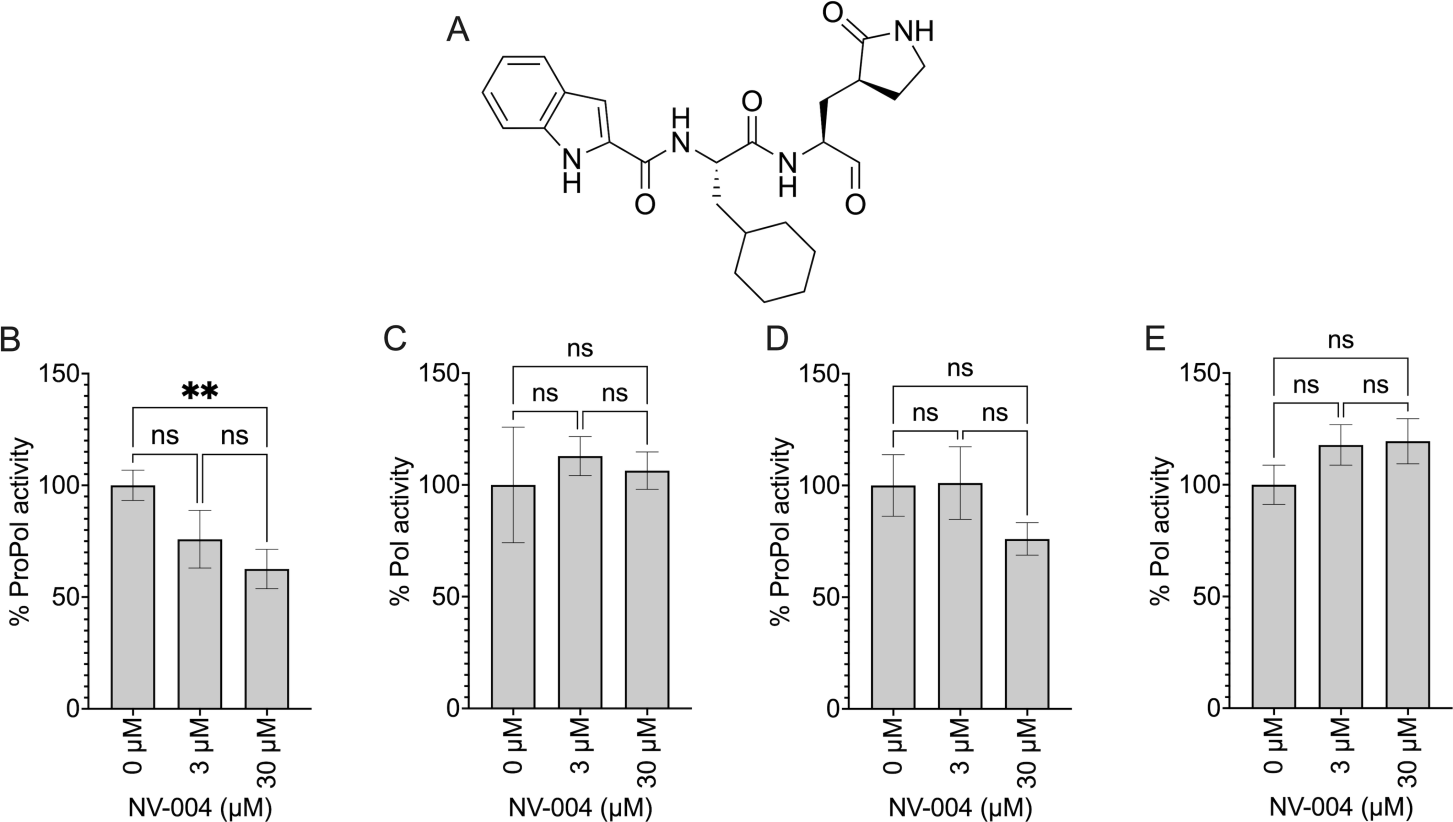
Inhibitory effect of NV-004 on HuNV polymerase activity. (**A**) Schematic of NV-004 peptidomimetic protease inhibitor. (**B**) GI ProPol, (**C**) GI Pol, (**D**) GII ProPol, and (**E**) GII Pol purified from *E. coli* were incubated with NV-004, and *de novo* polymerase activity was monitored by the formation of dsRNA from a poly(C) template. Polymerase activity of ProPol or Pol was normalized to the 0-µM NV-004 control. Results represent the mean and SD for three independent experiments with triplicate reactions for each measurement point. Data were analyzed by one-way ANOVA, ns = *P* > 0.05 and **P < 0.01.

As ProPol is a multi-functional enzyme with both protease and polymerase activities, we next investigated whether inhibition of protease activity can influence polymerase activity. NV-004, a peptidomimetic antiviral that inhibits protease activity of 3CL proteases, including norovirus Pro (IC_50_ 0.37 µM) (45, 46), was incubated with GI and GII ProPol or Pol and the *de novo* polymerase activity measured (Fig. 7). The mature GI and GII Pol showed no decrease in polymerase activity in the presence of NV-004 (Fig. 7C and E). Incubation of GI ProPol with NV-004 showed a decrease in polymerase activity. Particularly with 30 µM NV-004, activity was significantly decreased to 62% compared with a 0-µM control (Fig. 7B). Although not statistically significant, a similar trend was observed for GII ProPol, with polymerase activity decreased to 76% in the presence of 30 µM NV-004 (Fig. 7D). These results indicate that inhibition of protease activity by NV-004 has a modest inhibitory effect on polymerase activity of ProPol when present at high concentrations.

**Fig 8:**
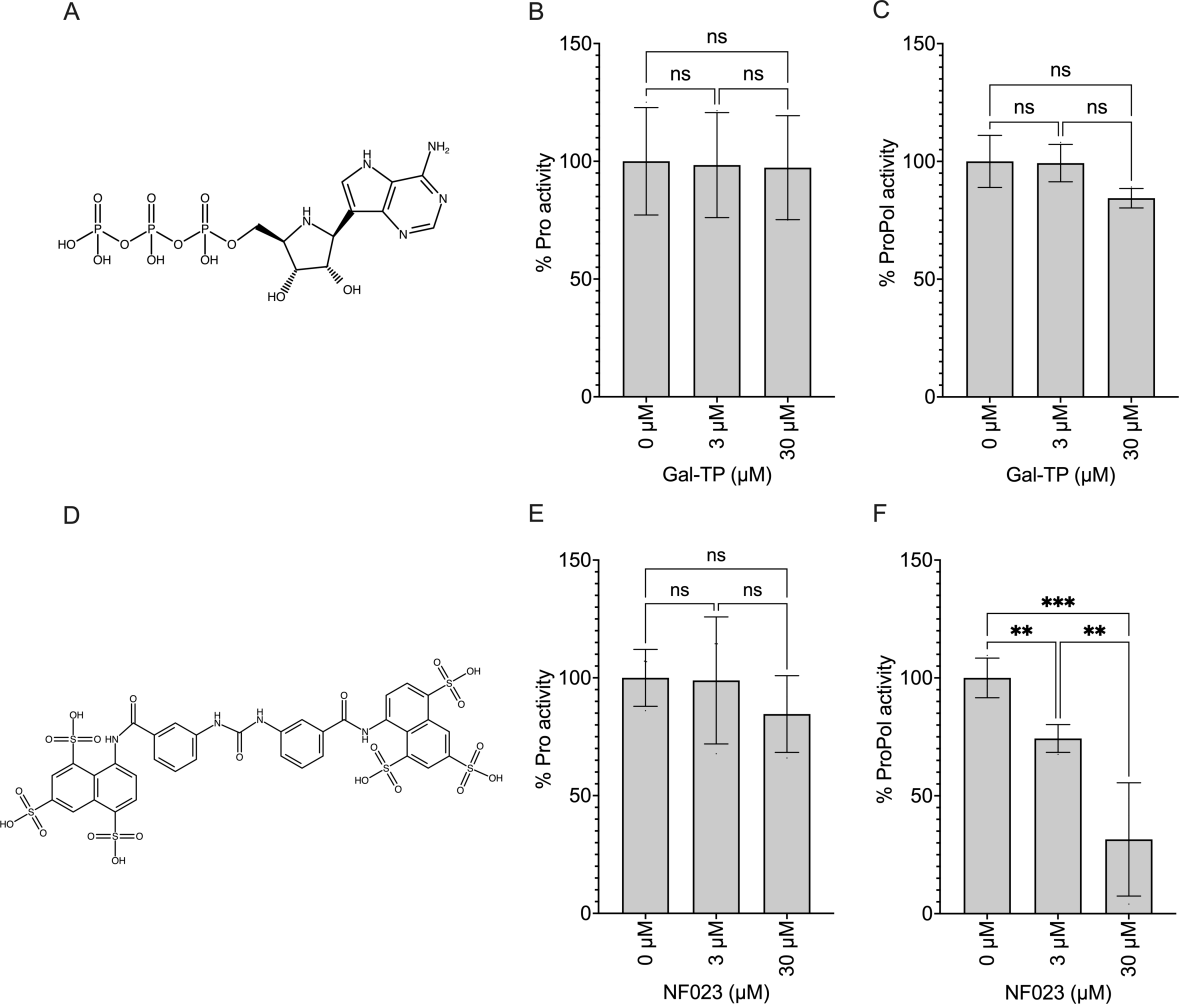
Inhibitory effect of Gal-TP and NF023 on protease activity of *E. coli*-purified GII HuNV Pro and ProPol. (**A and D**) Schematics of Gal-TP and NF023 polymerase inhibitors. (**B and C**) Protease activity of GII Pro and ProPol in the presence of Gal-TP. (**E and F**) Protease activity of GII Pro and ProPol in the presence of NF023. Protease activity was normalized to the 0-µM inhibitor control. Results represent the mean and SD for three independent experiments with triplicate reactions for each measurement point. Data were analyzed by one-way ANOVA, ns = *P* > 0.05, **P < 0.01, and ***P < 0.001.

Finally, we investigated whether inhibition of polymerase activity influences protease activity (Fig. 8). Incubation of the nucleotide inhibitor Gal-TP with GII Pro or ProPol did not affect protease activity to a level of significance, although there was an observable decrease in ProPol activity with 30 µM Gal-TP (Fig. 8B and C). As the fluorescence of PPNDS interfered with the protease assay, the non-nucleotide HuNV polymerase inhibitor NF023 was used as an alternative (47). The protease activity of GII Pro was not affected by NF023. However, protease activity of GII ProPol was significantly decreased to 74% and 45% with 3 µM and 30 µM NF023, respectively (Fig. 8E and F), showing that inhibition of polymerase activity with NF023 can decrease ProPol protease activity.

## DISCUSSION

The protease activity of mature and precursor norovirus proteases has been well studied; however, the polymerase function of norovirus ProPol has received limited attention. This study further characterizes the polymerase activity of GI and GII HuNV enzymes in the precursor and mature states.

There were differences in template preferences of GI and GII polymerases with homopolymeric RNA. This could influence replication of the virus and, in-part, explain the prolonged and severe symptoms and higher fecal load associated with GII.4 virus infections (48–51).

Distinct differences were observed in the *de novo* template utilization between ProPol and Pol, particularly for GII enzymes. In *de novo* polymerase assays, GII Pol showed activity with poly(C) and poly(U) RNA templates but was not active with a poly(A) template. These results are consistent with the literature, except for activity with a poly(U) template which previously could not be detected in a *de novo* polymerase reaction using 50 µM of ATP (26, 52, 53). In our experiments, poly(U) polymerase activity was apparent at concentrations of ATP greater than 50 µM and a *K*_m_ for ATP of ~480 µM is consistent with concentrations available in the cell (54, 55). In contrast to Pol, we have shown that GII ProPol can utilize poly(C), poly(U), and poly(A) templates and displays equal or superior kinetic parameters to Pol, apart from the *k*_cat_ for GTP.

Kinetic experiments with poly(U) RNA template or ATP produced curves with *R*^2^ values < 0.9 when fit with the Michaelis-Menten equation. Alternatively, the data can be fitted with an allosteric sigmoidal model that gives *R*^2^ values > 0.95 that could be indicative of allosteric interactions. Using this model did not change the trend for the catalytic parameters between ProPol and Pol, for example, the *K*_m_ as estimated by half *V*_max_ was 6.8 ng/µL for GII ProPol with poly(U) (7.9 ng/µL using the Michaelis-Menten equation). We did not see evidence of allosteric interactions with other RNA templates or nucleotides, nor did we see evidence of ProPol multimerization by cryo-EM.

Polymerase assays with poly(G) RNA showed negligible change of fluorescence in the presence of GI or GII polymerases. This indicates that norovirus polymerases do not function on poly(G) RNA or that the template is not conducive for measuring activity, possibly due to high background fluorescence or formation of RNA quadruplex structures (56–61). Regardless, poly(G) RNA was not pursued in kinetic experiments.

The impact of *de novo* polymerase activity and template preferences on initiation of GI and GII norovirus RNA replication is unknown as the precise mechanisms have not yet been clearly defined (26, 37, 53, 62). In one model, terminal transferase activity of norovirus Pol has been proposed to produce a poly(C) stretch on the 3′ terminus of the anti-genomic RNA (63). The poly(C) stretch could be used for *de novo* initiation by GI and/or GII norovirus ProPol and Pol during replication. In addition to poly(C) activity, our results demonstrate that GII ProPol can utilize poly(A) RNA as a *de novo* template. During viral replication, it is possible that GII ProPol replicates the negative-sense RNA in a primer-independent fashion using the poly(A) tail as a template from which to initiate synthesis. Future work should investigate the activity of ProPol with RNA sequences representing the viral genome to further characterize initiation of negative-strand synthesis.

To determine if structural changes might account for the differences in polymerase activity between ProPol and Pol, the structure of the polymerase domain was resolved by cryo-EM to 2.6 Å. In the consensus map, the density for the protease domain and linker region through to residue 6 of the polymerase domain could not be resolved. The interdomain region is flexible, and we hypothesize that this results in large degrees of freedom of the protease domain relative to the polymerase domain and “classing out” of the protease during image processing and 3D reconstruction. Other explanations, such as unfavorable interactions at the air-water interface during vitrification or proteolytic cleavage of the linker, were considered less likely due to a lack of preferential particle orientation and evidence that the sample was intact at the time of freezing (Fig. S3). Regardless, the ProPol structure presented in this paper represents the first structure of the norovirus polymerase domain in the context of a ProPol molecule.

The polymerase domain of GII.4 norovirus ProPol was solved in the absence of substrates but occupied with a metal cation. The active site did not contain unexplained densities within the cryo-EM map suggesting RNA was not co-purified with the protein. A modest shift was observed in the α-helix loop of Motif B, which is rather distant (~8 Å) from the nearest residue of the B-site and is on the opposite side of the internal compartment of the polymerase from the A-site. Motif B in RNA polymerases is involved in nucleotide recognition, coordinating the correct positioning of the sugar in the ribose-binding pocket, as well as template coordination and primer binding (64, 65). Although this structure is ligand free, the rearrangement of the loop is similar to the arrangement observed in PPNDS-bound mature Pol (33). Other than Motif B, the palm domain does not exhibit the variation that might be expected given the differences in activity and substrate specificity observed between the mature and precursor proteins. It may be that structural variations arise only once ProPol has progressed to a later state of catalysis or when bound to a substrate, nucleotide triphosphate (NTP) and/or VPg molecule(s). To this end, we expect a ProPol structure captured in a state of catalysis or bound with ligand or substrate, would likely be informative with regard to the difference in catalytic efficiency, and provide insights on the importance of these structural changes for viral replication.

The norovirus polymerase and protease represent major targets for antiviral development due to their essential role in the viral lifecycle. Both Gal-TP and PPNDS inhibited activity of ProPol and Pol, suggesting both enzymes can be targeted by a single antiviral albeit with differing efficacy. For GII Pol, IC_50_ results for PPNDS have been reported to fall in the range of 0.45–1.5 µM, consistent with our results (34, 36, 41). Interestingly, PPNDS was ~7-fold more effective against GII Pol than GII ProPol but the reason for this discrepancy is not clear. Comparison of unliganded GII ProPol and GII Pol structures shows that there are not any major structural changes in the B-site within the thumb domain, where PPNDS binds (33, 34). Interestingly, GI Pol displayed an IC_50_ of 3.4 µM with PPNDS, suggesting that the inhibitor may not be as effective against GI HuNV compared with GII.

In the context of ProPol, inhibition of one enzymatic activity, whether protease or polymerase, can affect the other activity. One explanation is that the binding of antivirals in the active site impacts the structural flexibility of the protease and polymerase domains relative to the other. NV-004 impacts the structure of GII.4 Pro, specifically the βeII-βfII, βbII-βcII, and βcII-βdII loops (46), and if similar changes occurred with ProPol, this could affect the structure of the polymerase domain.

Alternatively, an antiviral in the active site could influence substrate interactions of ProPol thereby impacting enzymatic functions. Picornavirus 3CD protein binds the 5′ cloverleaf RNA through the RNA binding region of the protease domain to correctly position the polymerase for replication (66, 67). Binding of NV-004 in the protease active site of ProPol could affect the RNA binding potential of the protease domain and result in decreased polymerase activity through a similar mechanism. Overall, the results with NV-004 and NF023 indicate that inhibition of protease or polymerase activity in the context of ProPol can be inhibitory to the alternate enzyme activity that is consistent with interplay between the domains.

In summary, this work shows that HuNV ProPol is an enzyme with overlapping but distinct recognition and utilization of substrates compared with mature Pol. The cryo-EM structure of the polymerase domain of GII ProPol indicates that the differences in activity are not reflected by the unliganded relaxed state structure of the enzyme. Despite the similarities in activity and structure, ProPol and Pol had differing responses to both Gal-TP and PPNDS, with higher IC_50_ values for ProPol compared with Pol. Together, these results indicate that ProPol and Pol play different but essential roles and implicate ProPol as an important target for antiviral testing.

## MATERIALS AND METHODS

### Compounds

The norovirus polymerase inhibitors, pyridoxal‐5′‐phosphate‐6‐(2′‐naphthylazo‐6′‐nitro‐4′,8′‐disulfonate) tetrasodium salt (PPNDS) and 8,8′-[carbonylbis(imino-3,1-phenylenecarbonylimino)]bis-1,3,5-naphthalene-trisulphonic acid (NF023), were sourced from Tocris Bioscience (Minneapolis, MN, USA) and MedChemExpress (Monmouth Junction, NJ, USA), respectively. Gal-TP was synthesized by the Ferrier Research Institute, Victoria University of Wellington, as described in reference (68). NV-004, a known SARS-CoV-2 mPro inhibitor (45), was synthesized by the University of Auckland as described in (46).

### Construction of norovirus ProPol and Pol plasmids

Synthetic genes encoding for HuNV GI Southampton and GII.4 Sydney 2012 ProPol and Pol proteins were obtained from GenBank (accession numbers L07418.1 and JX459908, respectively) and synthesized by GenScript (Piscataway, NJ, USA). ProPol sequences were modified to contain a mutation, E181A, at the Pro/Pol junction to prevent protease cleavage into the mature proteins (11, 17).

For expression of scarless proteins, the Expresso Rhamnose SUMO Cloning and Expression System was utilized (Lucigen, Middleton, WI, USA). Construction of plasmids for expression of HuNV ProPol and Pol proteins with an N-terminal His_6_ tag and SUMO fusion protein was performed according to the manufacturer’s instructions (Lucigen). Briefly, flanking 5′ and 3′ SUMO sequences complementary to the pRham N-His SUMO Kan vector (pRham) were introduced to gene sequences by PCR (Table 4). The PCR products along with pre-processed pRham plasmid were co-transformed into *E. coli* for homologous recombination to form the SUMO_Pol or SUMO_ProPol fusion construct. All constructs were confirmed by sequencing.

**TABLE 4:** Sequences of primers for generation of pRham SUMO_ProPol and SUMO_Pol plasmids

Construct	Primer sequence (5′−3′)^*a*^
SUMO_GII.4 ProPol	F: CGCGAACAGATTGGAGGTGCACCGCCTTCTATTTGGTCCCGTA R: GTGGCGGCCGCTCTATTATTCCACACCATCCTCGTTCACG
SUMO_GII.4 Pol	F: CGCGAACAGATTGGAGGTGGAGGAGACTCAAAGGGTACTT R: GTGGCGGCCGCTCTATTATTCCACACCATCCTCGTTCACG
SUMO_GI ProPol	F: CGCGAACAGATTGGAGGTGCACCTCCTACGTTGTGGT R: GTGGCGGCCGCTCTATTAAACACCGTCATCATTAACAAAC
SUMO_GI Pol	F: CGCGAACAGATTGGAGGTGGTGGCGATAAAGGTCACTA R: GTGGCGGCCGCTCTATTAAACACCGTCATCATTAACAAAC

^*a*^ Homologous sequences for SUMO recombination are underlined; F indicates forward primers, and R indicates reverse primers.

### Expression and purification of SUMO fusion proteins for enzymatic analyses

*E. coli* cultures containing SUMO_Pol or SUMO_ProPol constructs were grown in terrific broth until an OD_600_ of approximately 0.5 was reached and expression of SUMO fusion proteins was induced with 0.2% L-rhamnose. Cultures were grown for a further 6 h at 37°C, and the bacterial pellet was collected by centrifugation. The cells were lysed by sonication in 50 mM HEPES pH 7.5, 300 mM NaCl, 15 mM imidazole, 5 mM β-mercaptoethanol, and 20% glycerol. The soluble fraction was collected following centrifugation at 14,000 × *g* and incubated with Ni-NTA agarose beads (Qiagen, Hilden, Germany), and the bound protein was collected by gravity flow column (Pierce, Waltham, MA, USA). Proteins were eluted in 50 mM HEPES pH 7.5, 150 mM NaCl, 150 mM imidazole, and 20% glycerol. For SUMO fusion partner removal, purified proteins were buffer exchanged using Amicon ultra-centrifugal filter units (Millipore, Burlington, MA, USA) into 50 mM HEPES pH 7.5, 150 mM NaCl, 10% glycerol, and 2 mM DTT. Cleavage reactions were incubated overnight at 4°C with 0.5 U/mg (SUMO_Pol) or 5 U/mg (SUMO_ProPol) SUMO Express Protease (Lucigen). The reaction mixture was incubated with Ni-NTA agarose to remove the His_6_ tagged SUMO peptide and SUMO protease. Purified protein was collected, quantified by densitometry on SDS-PAGE gels with BSA as a protein standard, and stored at −80°C in 50 mM HEPES pH 7.5, 150 mM NaCl, and 50% glycerol. Protein identity was confirmed by mass spectrometry (Centre for Protein Research, University of Otago, NZ). Three separate batches of protein were produced and used as biological replicates in enzyme assays. Expression and purification of GII.4 Pro were performed as described previously (46).

### Expression of H_6_ProPol for cryo-EM

GII.4 Sydney 2012 ProPol was expressed using the commercial recombinant baculovirus system Flashback Ultra (Oxford Expression Technologies, Oxford, UK). The expression construct contained an N-terminal His_6_ tag with flexible linker (DYDIPTT) followed by ProPol, termed H_6_ProPol to differentiate from SUMO_ProPol. *Trichoplusia ni* insect cells in suspension culture were infected with recombinant baculovirus expressing H_6_ProPol at a multiplicity of infection of 1 and incubated at 27°C with 125 RPM for 3 days. Cells were lysed in buffer containing 50 mM HEPES pH 8, 300 mM NaCl, and 1% Triton X-100. Soluble protein was purified as described for SUMO_ProPol, except that the elution buffer did not contain any glycerol. Samples were further purified by size exclusion chromatography using an S12 10/300 column (Cytiva, Marlborough, MA, USA) on an Äkta Purifier 10 fast protein liquid chromatography system (GE Life Sciences, Chicago, IL, USA), and the central fraction of the elution peak was collected and diluted in 50 mM HEPES pH 7.5, 150 mM NaCl, 30 mM imidazole, 2 mM MgCl_2_, and 2 mM DTT to 1 mg/mL.

### Protease assay with Pro and ProPol

A fluorescent resonance energy transfer assay was used to measure the activities of Pro and ProPol as described in reference (46). The peptide substrate, 5(6)-carboxyfluorescein (FAM)-LGDYEL**QG**PEDLAK-Dabcyl utilized in reactions, was based on the NS1-2/NS3 cleavage site of HuNV GII.4 Sydney 2012, and the scissile bond is shown in bold.

### Kinetics of ProPol and Pol activity

*De novo* polymerase activity was measured by monitoring the formation of dsRNA from a single-stranded homopolymeric RNA template using PicoGreen as described previously (69). Optimal pH (range of 7.0–8.0), concentrations of MnCl_2_ (2.5 mM), and DTT (5 mM) for HuNV were previously established (17, 52). Concentrations of NaCl in the final polymerase reaction were maintained below 10 mM (17).

Standard reactions were performed in 25 µL, containing 5 mM DTT, 2.5 mM MnCl_2_, 20 ng/µL RNA template, 0.5 mM NTP in 20 mM Tris pH 7.5. Reactions were initiated with the indicated concentration of enzyme and allowed to proceed for a set period at 37°C before being terminated with 10 mM EDTA. PicoGreen (1:350 dilution) (Invitrogen, Waltham, MA, USA) was added to bind the dsRNA, and fluorescence was measured on a Varioskan Flash (Thermo Fisher Scientific, Waltham, MA, USA) plate reader with excitation at 480 nm and emission at 520 nm.

Kinetics of nucleotide incorporation by polymerase proteins was analyzed by titrating NTP concentration under standard reaction conditions for the linear time period of the enzyme. Kinetics of RNA template utilization was analyzed in the same manner but with titration of the homopolymeric template. The velocity of the reaction against substrate concentration was plotted, and the kinetic parameters were determined by non-linear regression using the Michaelis-Menten model in GraphPad Prism version 9.2.0 (La Jolla, CA, USA). All enzyme kinetics was performed with three independent preparations of protein, with triplicate wells for each replicate.

### Enzymatic IC_50_ assay

For the determination of IC_50_ values, titrations of the test compound (0–10 μM for PPNDS and 1.2–1200 μM for Gal-TP) or a vehicle control were incubated with the enzyme, for 20 min in 20 mM Tris pH 7.5, 5 mM DTT, and 2.5 mM MnCl_2_. Substrate, at a concentration below the calculated *K*_m_ values, was added and incubated for the indicated time. Reactions were stopped with EDTA, dsRNA stained with PicoGreen, and fluorescence measured on a Victor Nivo (PerkinElmer, Shelton, CT, USA) plate reader. IC_50_ values were determined by non-linear regression using the log (inhibitor) vs. normalized response—variable slope model in GraphPad Prism. In the experiments, a maximal PPNDS concentration of 10 µM was used as higher concentrations fluoresced at the same wavelength as PicoGreen.

Inhibition of polymerase activity by the protease inhibitor NV-004 (46) was determined by incubating enzyme with an inhibitor at 0, 3, and 30 µM for 20 min. Substrate was added to the reaction, and the percentage activity was calculated relative to the 0 µM control.

### Cryo-EM sample preparation and data collection

UltrAUFoil R1.2/1.3 300 mesh gold grids (Electron Microscopy Sciences, PA, USA) were glow discharged for 120 s at 15 mA in a Pelco Easiglow Glow Discharge Cleaning System (Pelco, CA, USA) before 3 µL of sample was applied, and the grids were plunge frozen into liquid ethane using a FEI Mark IV Vitrobot (Thermo Scientific, Waltham, MA, USA). Data were collected using EPU software on a FEI Titan Krios G3i (Thermo Scientific) operating at a voltage of 300 kV with a Gatan BioQuantum energy filter and a K2 Gatan camera system. Exposures were collected at a nominal magnification of 215,000× for 5 s with an accumulated electron dose of 80 e/ Å^2^ at a pixel size of 0.66 (Å/pix). Exposures were collected with a range of defoci from −2.0 to −1.0 µm. Approximately 9,300 exposures were obtained. Statistics for data collection and refinement are listed in Table 5.

**TABLE 5:** Cryo-EM data collection, refinement, and validation statistics for GII.4 Sydney 2012 ProPol polymerase domain

Data collection and processing	
Magnification	215K
Voltage (kV)	300
Electron exposure (e/Å^2^)	80
Spherical aberration (mm)	2.7
Defocus range (μm)	−2.0 to −1.0
Pixel size (Å/pix)	0.66
Initial particle images (no.)	>5,000,000
Final particle images (no.)	997,801
Map resolution (Å)	2.6
Refinement	
Initial model used (PDB code)	4LQ3
Map sharpening B factor (Å)	52.87
Bond lengths (Å)	0.012
Validation	
MolProbity score	1.18
EMRinger score	2.35
Clashscore	3.79
Poor rotamers (%)	0 (0)
Ramachandran plot	
Favored (%)	97.98
Allowed (%)	1.8
Disallowed (%)	0.20
C-beta outliers (%)	0

### Cryo-EM data processing and model building

All data processing (motion correction, 2D and 3D classification, and *ab initio* reconstruction) was performed in cryoSPARC v. 3.0 (70) (Fig. S4). Particles were picked using the blob picker job and submitted to 2D classing (Fig. S6). Classes capturing multiple viewing directions were used as templates in a template picking job, and the particles were sorted via iterative 2D classification to produce well-defined classes with visible secondary features. Roughly 900,000 particles were selected after 2D classification and used for *ab initio* reconstruction into three classes. The best 3D class was selected based on the broadest range of particle orientations and was used as a 3D volume template for several rounds of homogeneous and non-uniform refinement with per-group CTF and per-particle defocus estimation features switched on. Refinement was performed iteratively until resolution no longer improved. A 3D variability job was performed to identify local motion at the Pro-Pol linker but ultimately did not allow for the resolution of the Pro domain. Model building was performed by manual and automated refinement residue by residue using real-space refinement in Phenix (71, 72), manual refinement in COOT (73), and ISOLDE v. 1.3 (74) and validated in Molprobity (75). The GII.4 Dresden polymerase structure (PDB: 4LQ3) was used as an initial model for the polymerase domain, mutated to the amino acid identity of GII.4 Sydney 2012 Pol, fit into the map using UCSF ChimeraX (76), and refined into the cryo-EM density map.

## Data Availability

Maps and models for HuNV Sydney 2012 ProPol polymerase domain have been deposited in the Protein Data Bank and the Electron Microscopy Data Bank (EMDB), under accession codes 9BI9 and EMD-44577, respectively.

## SUPPLEMENTAL MATERIAL

Supplemental figures jvi.01193-24-s0001.pdf Figures S1 to S6.

ASM does not own the copyrights to Supplemental Material that may be linked to, or accessed through, an article. The authors have granted ASM a non-exclusive, world-wide license to publish the Supplemental Material files. Please contact the corresponding author directly for reuse.
