# Supplementary material for: Activity and cryo-EM structure of the polymerase domain of the human norovirus ProPol precursor
Source: J Virol. 2024 Oct 30;98(11):e01193-24. doi: 10.1128/jvi.01193-24 (PMC11575396; doi:10.1128/jvi.01193-24)
Supplement: Supplemental figures — Figures S1 to S6. [file jvi.01193-24-s0001.pdf]

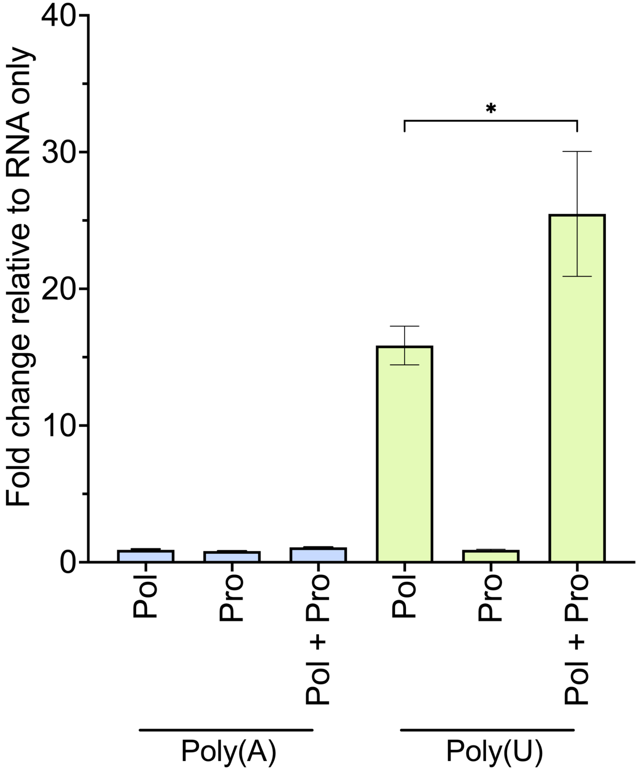


Figure S1: *De novo* polymerase activity of GII Pol in the presence of GII Pro. Polymerase activity with of 1 μM of each enzyme, 20 ng/μl template, 0.5 mM NTP for 80 minutes. Fold change relative to an RNA only control was calculated. Results represent the mean and SD of three independent reactions. Data was analysed by two-tailed unpaired t-test, * $P < 0.05$.

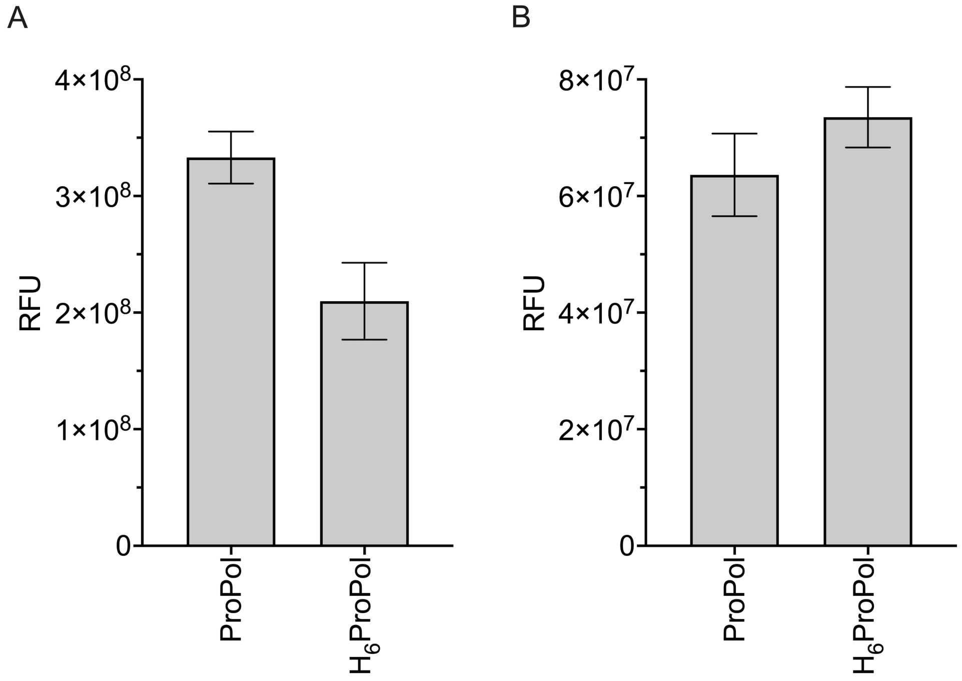


Figure S2: (A) Protease activity of GII ProPol and $H_6$ProPol. Each protein (2 μM) was incubated with 10 μM of FRET peptide substrate for 10 minutes and fluorescence (RFU) was measured. (B) Polymerase activity of GII ProPol and $H_6$ProPol. Each protein (35 nM) was incubated in a standard polymerase assay for 10 mins and the fluorescence measured. Data represents the mean and SD of three biological repeats in technical triplicate.

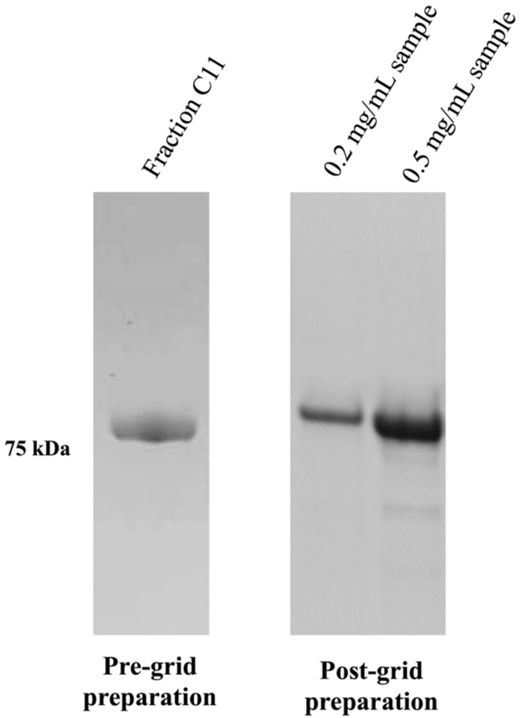


Figure S3: Comparison of $H_6$ProPol protein before and after cyro-EM grid preparation. $H_6$ProPol was run on an 4-20% pre-cast SDS-PAGE gel (Bio-Rad) to confirm homogeneity. (A) SEC sample of protein prior to grid preparation. (B) Samples at two concentrations after preparation of cryo-EM grids.

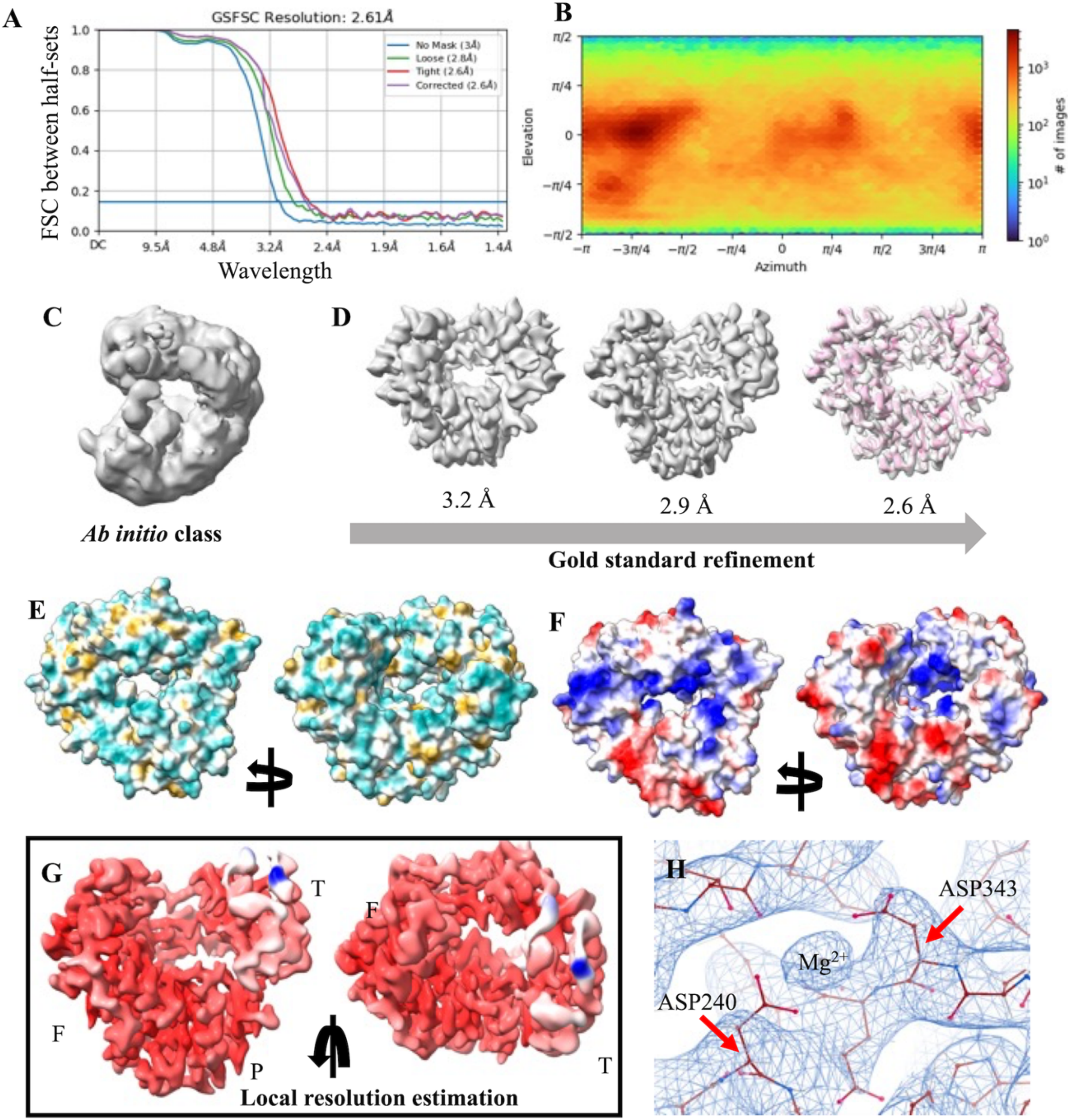
A
GSFSC Resolution: 2.61Å
FSC between half-sets
Wavelength
No Mask (3Å)
Loose (2.8Å)
Tight (2.6Å)
Corrected (2.6Å)
DC
9.5Å
4.8Å
3.2Å
2.4Å
1.9Å
1.6Å
1.4Å
B
Elevation
Azimuth
# of images
C
Ab initio class
D
3.2 Å
2.9 Å
2.6 Å
Gold standard refinement
E
F
G
T
F
F
P
T
Local resolution estimation
H
ASP343
Mg2+
ASP240

Figure S4: Structural refinement of the Pol domain of $H_6$ProPol. (A) Gold standard Fourier shell correlation (GSFSC) plot produced from cryoSPARC for the final consensus map of ProPol. (B) Particle orientation distribution plot. The diffuse orange and red indicates there was good distribution of particle views within the data set. (C) An initial class produced from an *ab initio* reconstruction job prior to homogeneous refinement. (D) Improvement in map resolution as gold standard refinement is iteratively performed. The final map is displayed with 50% transparency and the model is nested inside. (E) The model is displayed in smoothed van der Waals molecular surface with hydrophobicity calculated in UCSF ChimeraX (cyan for most hydrophilic potentials and gold for most hydrophobic potentials). (F) Electrostatic potential calculated in UCSF ChimeraX is coloured red, white and blue for negative, neutral and positive charges, respectively. (G) Sharpened 2.61 Å map filtered by local resolution (red = 2.6 Å, white = 2.8 Å, blue = 3 Å). Local resolution in the palm (P) and fingers (F) domains is high relative to the tip of the thumb domain (T). (E) The polymerase model (red sticks) was refined into the map electron density (blue netting) in COOT. Two aspartate residues, 240 and 343, are shown coordinating the $Mg^{2+}$ divalent metal cation in the active site cleft.

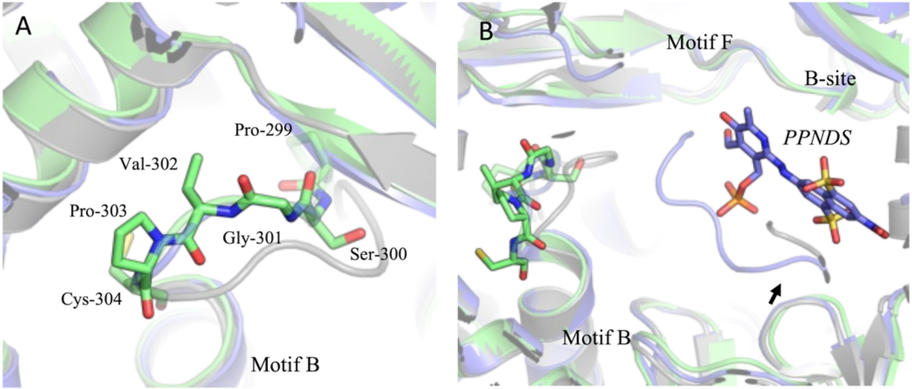


Figure S5: Motif-B of unliganded Sydney 2012 ProPol (green) overlayed with unliganded Dresden Pol (PDB: 4LQ3) (grey) and PPNDS-bound Dresden Pol (PDB: 2B43) (purple). (A) Overlay of the α-helix loop in Motif B. A cropped view of Motif B within the map density is visible in the top right corner. (B) Overlay depicting the spatial relationship between Motif B and the B-site where PPNDS is bound in mature Pol (PDB: 4LQ3). Black arrow indicates the C-terminal tail of unliganded and PPNDS-bound Pol in the template entry channel.

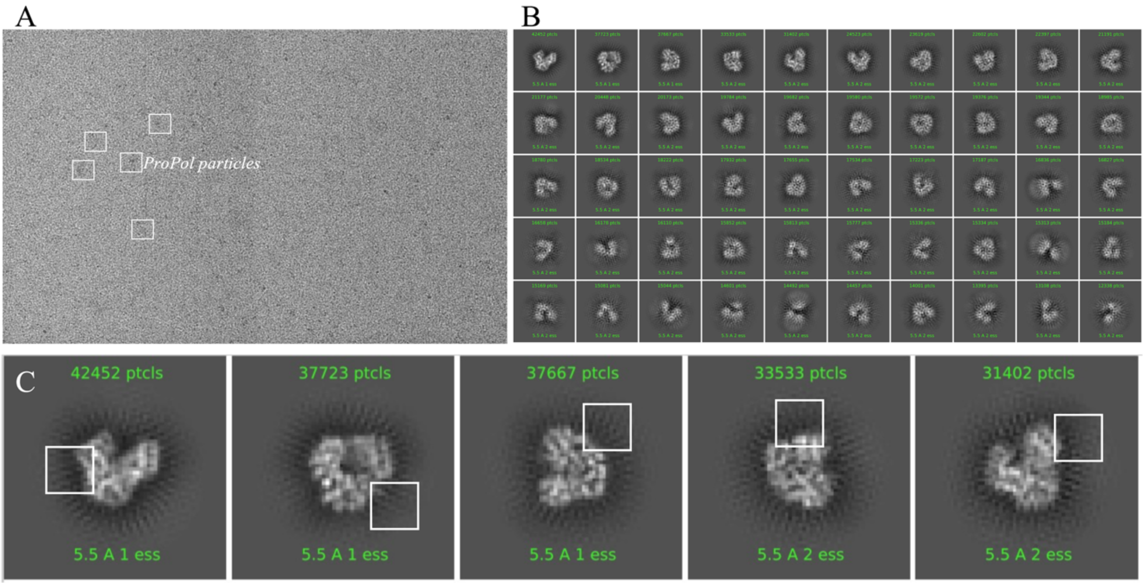


Figure S6: $H_6$ProPol cryo-EM 2D classes produced in CryoSPARC. (A) A representative micrograph demonstrates the low signal-to-noise ratio recorded in the images. $H_6$ProPol particles are indicated in white boxes. (B) 2D class averages were produced in CryoSPARC from a collection of ~1,000,000 particles to a minimum resolution cut-off of 5.5 Å. Classes show a single monomeric polymerase domain from several orientations including top and side views. (C) The area where density for the protease domain would roughly be expected is indicated by a white box. There is no clear or obvious smearing or blurring along the edges of classes, as might be expected for a flexible domain, indicating that the protease domain has been classed out of the averages likely do to the very small size of the protease.
